# Gait Characteristics and Brain Activity in Parkinson’s Disease with Concomitant Postural Abnormalities

**DOI:** 10.14336/AD.2019.0929

**Published:** 2020-07-23

**Authors:** Meng-sha Yao, Li-che Zhou, Yu-yan Tan, Hong Jiang, Zhi-chun Chen, Lin Zhu, Ning-di Luo, Quan-zhou Wu, Wen-yan Kang, Jun Liu

**Affiliations:** ^1^Department of Neurology & Collaborative Innovation Center for Brain Science, Ruijin Hospital, Shanghai Jiao Tong University School of Medicine, Shanghai, China.; ^2^Department of Neurosurgery, Ruijin Hospital, Shanghai Jiao Tong University School of Medicine, Shanghai, China.; ^3^State Key Laboratory of ISN, School of Computer Science and Technology, Xidian University, Xi'an, Shanxi Province, China.; ^4^Department of Neurology, Ruijin Hospital North, Shanghai Jiao Tong University School of Medicine, Shanghai, China.

**Keywords:** Parkinson’s disease, postural abnormality, camptocormia, Pisa syndrome, gait

## Abstract

To explore the underlying pathogenic mechanism of Parkinson’s disease (PD) with concomitant postural abnormalities (PDPA) through the relationship between its gait and brain function characteristics. PD patients from the neurology outpatient clinic at Ruijin Hospital were recruited and grouped according to whether postural abnormalities (including camptocormia and Pisa syndrome) were present. PD-related scale assessments, three-dimensional gait tests and brain resting-state functional magnetic imaging were performed and analyzed. The gait characteristics independently associated with PDPA were decreased pelvic obliquity angle and progressive downward movement of the center of mass during walking. PDPA features included decreased functional connectivity between the left insula and bilateral supplementary motor area, which was significantly correlated with reduced Berg Balance Scale scores. Functional connectivity between the right insula and bilateral middle frontal gyrus was decreased and significantly correlated with a decreased pelvic obliquity angle and poor performance on the Timed Up and Go test. Moreover, through diffusion tensor imaging analysis, the average fractional anisotropy value of the fibers connecting the left insula and left supplementary motor area was shown to be decreased in PDPA. There is decreased functional connectivity among the insula, supplementary motor area and middle frontal gyrus with structural abnormalities between the left insula and the left supplementary motor area; these changes in brain connectivity are probably among the causes of gait dysfunction in PDPA and provide some clues regarding the pathogenic mechanisms of PDPA.

Postural abnormality increases in prevalence as Parkinson’s disease (PD) progresses; the combination of these conditions is termed PD with concomitant postural abnormalities (PDPA). Camptocormia (CC) and Pisa syndrome (PS) are the most common causes of significant postural instability and increase the risks of falls, long-term bedridden status and death [1]. There is no internationally recognized standard for the flexion angles that constitute CC and PS; the former is defined as voluntarily anterior thoracolumbar flexion of at least 30° or 45°, and the latter means voluntarily lateral flexion of the trunk by at least 10° or 15° [1-4]. Both conditions can be completely restored to normal in the supine position or by passive efforts but cannot be corrected under the patient’s own power during extended sitting or walking. The reported prevalence of postural abnormalities in PD patients varies across different ranges, with that of CC being 4.1-17.7% [5] and that of PS 1.9-12.2% [3].

However, the effectiveness of current therapies for PDPA is fairly limited because its pathogenic mechanism remains unclear, and both central and peripheral factors are involved. The main central hypotheses include an imbalance of related neurotransmitters and damage to the basal ganglia, proprioceptive dysfunction and vestibular dysfunction [6-8]. The main peripheral hypotheses refer to functional muscular disorders [9, 10]. Central pathological changes are considered the initial factors, while peripheral lesions are secondary and interact with the central factors to exacerbate postural abnormalities. Some studies demonstrated a correlation of the insula [11], pedunculopontine nucleus [12], and frontal lobes [13] with postural control, which may provide a reasonable basis for the exploration of postural control in PDPA. Among these affected brain areas, areas that are relatively anatomically superficial, such as the frontal lobes and the insula, might be potential therapeutic targets for noninvasive interventions, such as transcranial magnetic stimulation (TMS).

PDPA patients are prone to gait dysfunctions, including reduced walking speed, a decreased range of joint flexion angles, impaired balance and poor performance on complicated gait tasks [14]. However, few studies have specifically focused on gait function in PDPA, and no study has explained the pathogenic mechanism of gait dysfunctions in PDPA. Resting-state functional magnetic resonance imaging (rs-fMRI) is a noninvasive brain imaging technique that has been used in many neurological and psychiatric diseases for the early detection of functional abnormalities. Functional connectivity (FC) analysis and structural fractional anisotropy (FA) analysis are the most commonly used techniques [15, 16].

We hypothesized that some brain areas connecting the frontal lobes or insula in PDPA were abnormal and were involved in the underlying pathogenic causes, subsequently affecting the gait ability of these patients. Thus, we investigated the gait characteristics of PDPA using related scales and a three-dimensional (3D) gait test, followed by rs-fMRI to obtain resting fMRI and diffusion tensor imaging (DTI) parameters. Finally, the correlation between gait characteristics and brain FC was analyzed with structural evidence via FA comparison. The results could help explain the mechanism of PDPA, which will be useful for developing potential interventions.

## MATERIALS AND METHODS

### Patients

PD patients who visited the neurology outpatient clinic at Ruijin Hospital from February 2017 to September 2018 were recruited. The study was approved by the Ethics Committee of Ruijin Hospital, and all enrolled subjects signed informed consent forms for participation in this study and publication of relevant data.

PD patients were diagnosed according to the new edition of the Movement Disorder Society’s diagnostic guidelines [17]. Postural abnormalities include CC, defined as anterior thoracolumbar flexion over 30°, and PS, defined as lateral trunk flexion over 10°. Patients were excluded if they were unable to walk at least 10 meters continuously without any assistance or suffered other obvious disorders involving the nervous system or musculoskeletal system.

Those who completed the PD scale assessment, 3D gait test and brain rs-fMRI scanning were enrolled in the study. Subjects who had CC or PS (or both) were grouped in the abnormal posture group, referred to as the ‘PD + CCPS’ group, while the others were placed in the normal posture group, referred to as the ‘PD - CCPS’ group. The groups were matched by age, sex, body mass index (BMI), years of education, and duration of PD.

Thirty-seven subjects completed the 3D gait test; 16 in the PD + CCPS group and 21 in the PD - CCPS group underwent gait analyses. After we excluded those unwilling or unable to undergo MRI, 20 of these patients participated in the MRI scans, with 7 in the PD + CCPS group and 13 in the PD - CCPS group. Due to severely limited motor ability that might not allow safe completion of the gait tests during off-state, all assessments were performed 1-2 h after the subjects took their routine medication.

### Clinical evaluations

The sagittal angle between a vertical line and a line connecting the trochanter with the edge of the acromion was evaluated to define camptocormia[4, 18], and the coronal angle between a vertical line and a line passing through the C7 and L4 vertebrae was used to define PS [1, 19].

All enrolled subjects underwent basic information collection, including age, sex, BMI, years of education, disease duration, and currently stable anti-PD drug regimen. The motor symptoms of PD were evaluated via the Movement Disorder Society Unified Parkinson’s Disease Rating Scale (MDS-UPDRS) part III [20] score and Hoehn-Yahr stage [21]. The Mini-Mental State Examination (MMSE) [22] and the Montreal Cognitive Assessment (MoCA) [23] were used for cognitive evaluation. The Berg Balance Scale (BBS) [24] was used to assess balance ability. The Timed Up and Go (TUG) Test [25] was performed as a comprehensive evaluation of gait function.

The 3D gait test started by establishing a static model of the subject with 39 reflective markers bilaterally attached to the body surface to draw the outline of the human body. Then, the subject was required to walk straight back and forth along a 9-meter trajectory. A ten-camera 3D motion capture system (Vicon Motion Systems Ltd, UK) was applied to collect gait data. At least three valid data sets were collected after the subjects had practiced maintaining consistent performance.

The kinematic and dynamic parameters were recorded synchronously, with all the length-related parameters standardized by the subject’s height. The vertical movement track of the body's center of mass (CoM) was obtained by generating a scatter plot of the moving coordinates, and a trend line was used to determine whether the subject had progressive downward movement of the CoM while walking.

### MRI acquisition

MRI scans were performed using a 3.0 T Trio Siemens MRI scanner equipped with a 12-channel coil in the Functional Imaging Centre of the Institute of Neuroscience of the Chinese Academy of Science in Shanghai. T1-weighted imaging was obtained by 3D magnetization-prepared rapid acquisition gradient echo (MPRAGE), with the following parameters: sequence of 176 axial slices; slice thickness, 1.0 mm; flip angle, 9°; voxel size, 1.0*1.0*1.0 mm^3^; echo time (TE), 3 ms; repetition time (TR), 2300 ms; and inversion time (TI), 1000 ms. Data were obtained via echo-planar imaging (EPI) as follows: sequence of 32 axial slices; slice thickness, 4.0 mm; flip angle, 90°; voxel size, 3.8*3.8*4.0 mm^3^; TE, 30 ms; and TR, 2000 ms. DTI data were acquired with 42 slices; slice thickness, 3 mm; voxel size, 1.7*1.7*3.0 mm^3^; TE, 94 ms; TR, 6000 ms; b-values, 0, 1000 s/mm^2^; diffusion gradient directions, 30; and FOV, 220 mm. Patients were instructed to remain awake with their heads still, their eyes closed and no specific thoughts.

### FC analyses based on BOLD data

The rs-fMRI images were preprocessed with GRETNA (www.nitrc.org/projects/gretna; version 2.0), a network analysis toolbox run in MATLAB R2013b (The MathWorks, Natick, MA, USA) and based on graph theory[26]; in brief, the preprocessing steps were as follows[27]: removal of the first 10 time points, slice-timing correction, motion correction, spatial normalization to the Montreal Neurological Institute (MNI) template (resampling to 3*3*3 mm^3^ voxels), spatial Gaussian smoothing (4 mm FWHM), elimination of nonneuronal signal fluctuations, and bandpass filtering to eliminate low-frequency drift data and high-frequency physiological signal noise (0.01-0.1 Hz).

**Table 1:** Baseline information of PD patients enrolled in gait analyses.

	PD - CCPS (n=21)	PD + CCPS (n=16)	*p* value
**Age (y)**	64.7±4.5	69.0±8.4	0.079^a^
**Sex (female/male, n)**	11/10	8/8	0.886^b^
**BMI (kg/m^2^)**	23.3±3.7	23.9±3.1	0.616^a^
**Education (y)**	10.2±3.2	10.6±4.2	0.762^a^
**Disease duration (y)**	5.4±4.4	7.3±4.5	0.226^a^
**Hoehn-Yahr stage**	2.0(1.0, 2.0)	2.5(1.6, 3.0)	0.006^c^
**LEDD (mg)**	405.64±353.76^d^	724.10±276.66	0.006^a^
**MDS-UPDRS III**	29.0±12.2	39.4±12.3	0.023^a^
**MMSE**	28.0(24.5, 28.5)	27.0(26.0, 28.0)	0.844^c^
**MoCA**	22.7±3.9	22.2±5.4	0.757^a^

PD: Parkinson’s disease; CC: camptocormia; PS: Pisa syndrome; PD - CCPS: PD without CC or PS group; PD + CCPS: PD with CC or PS group; BMI: body mass index; LEDD: levodopa equal daily dose; MDS-UPDRS: Movement Disorder Society Unified Parkinson’s Disease Rating Scale; MMSE: Mini-Mental State Examination; MoCA: Montreal Cognitive Assessment. The values are expressed as the mean ± SD for normally distributed continuous variables, mean (interquartile range) for abnormally distributed continuous variables, and number for categorical variables.

a Independent samples t-test;

b Crosstabs;

c Mann-Whitney test.

d Data were unavailable for 1 patient. *p*<0.05 is considered statistically significant.

Previous studies using fMRI have demonstrated that some areas are associated with postural control, and to provide potential targets for future TMS intervention, the frontal lobes and insula were selected as regions of interest (ROIs) in this study [11, 13]. The WFU PickAtlas in Statistical Parametric Mapping (SPM12, www.fil.ion.ucl.ac.uk/spm/software/spm12) [28] was used to designate these ROIs in the Automated Anatomical Labeling (AAL) atlas. The correlation coefficients of the bilateral frontal lobe and insula with the whole brain were calculated, and the FC maps of the ROIs with the whole brain were obtained. Finally, Fisher’s r-to-z transformation was performed to convert the FC matrix to a z-value matrix for subsequent statistical analysis.

Data Processing and Analysis of Brain Imaging (DPABI_v3.1, http://rfmri.org/dpabi) [29] was used to conduct a two-sample *t-*test between the two groups for each ROI-voxel z-value matrix of FC (TFCE (Threshold-Free Cluster Enhancement) method for multiple comparison enhancement). Resting-State fMRI Data Analysis Toolkit Plus (RESTplus_v1.1, www.restfmri.net) [30] was used to analyze the correlation between the z-value matrixes of FC and clinical characteristics (AlphaSim method for multiple comparison correction, voxel-level *p* < 0.05 when cluster size > 85 voxels; refer to AlphaSim in AFNI, http://afni.nimh.nih.gov/pub/dist/doc/manual/AlphaSim).

**Table 2:** Baseline information of PD patients enrolled in MRI analyses.

	PD - CCPS (n=13)	PD + CCPS (n=7)	*p* value
**Age (y)**	65.0±5.4	69.7±8.2	0.138^a^
**Sex (female/male, n)**	6/7	4/3	1.000^b^
**BMI (kg/m^2^)**	23.12±4.48	23.79±3.44	0.735^a^
**Education (y)**	12.0(9.0, 13.0)	10.0(8.0, 14.0)	0.699^c^
**Disease duration (y)**	5.4±4.9	8.4±5.0	0.215^a^
**Hoehn-Yahr stage**	1.7±0.7	2.2±0.7	0.116^a^
**MDS-UPDRS III**	31.6±13.8	35.1±12.5	0.581^a^
**LEDD (mg)**	346.32±372.29^d^	606.07±213.81	0.111^a^
**MMSE**	28.0(26.0, 29.0)	27.0(25.0, 27.0)	0.056^c^
**MoCA**	23.9±3.0	20.6±6.6	0.140^a^

PD: Parkinson’s disease; CC: camptocormia; PS: Pisa syndrome; PD - CCPS: PD without CC or PS group; PD + CCPS: PD with CC or PS group; MRI: magnetic resonance imaging; BMI: body mass index; LEDD: levodopa equal daily dose; MDS-UPDRS: Movement Disorder Society Unified Parkinson’s Disease Rating Scale; MMSE: Mini-Mental State Examination; MoCA: Montreal Cognitive Assessment. The values are expressed as the mean ± SD for normally distributed continuous variables, mean (interquartile range) for abnormally distributed continuous variables, and number for categorical variables.

a Independent samples t-test;

b Crosstabs;

c Mann-Whitney test.

d Data were unavailable for 1 patient. *p*<0.05 is considered statistically significant.

### DTI structural connectivity analyses

After the data were resampled to 2*2*2 mm^3^ voxels, a mask for each subject was generated based on the image of the gradient sensitivity factor b=0 s/mm^2^. The brain was extracted by the Brain Extraction Tool (BET) of FSL (FMRIB’s Software Library, http://fsl.fmrib.ox.ac.uk/fsl; version 5.0.9; threshold set to 0.2) to eliminate the images of the scalp and skull[31]. The FDT (FMRIB’s Diffusion Toolbox) package in FSL was used to correct the head movement and eddy current of the NIFTI data of each subject [32]. FA images were obtained by calculating the eigenvalues λ1, λ2 and λ3 with the FDT toolbox.

Deterministic tractography was performed using the FACT (Fiber Assignment by Continuous Tracking) algorithm in PANDA (www.nitrc.org/projects/panda; version 1.3.1) [33] and ended at voxels with FA < 0.2 or a tract turning angle of < 45˚ [34]. With the AAL atlas being the path of brain parcellation, the average FA matrix was obtained across 90 regions. The FA value represents the anisotropic diffusion level of white matter in voxels, which is the most commonly used indicator for examining brain connectivity microstructure. Average FA values of fiber between brain regions that were found to be significantly valuable from the above FC analyses were selected for an independent samples *t*-test in SPSS 22.0 software with *p* < 0.05 considered to indicate statistical significance.

### Statistical analysis

For comparison of clinical evaluations between the two groups, statistical analysis was performed using SPSS 22.0 software. An independent samples *t*-test, the Mann-Whitney test, and the chi-squared test were used to compare the corresponding categories of variables between groups. Binary logistic regression was used for multivariate correlation analysis. *p*<0.05 was considered statistically significant.

**Figure 1.**
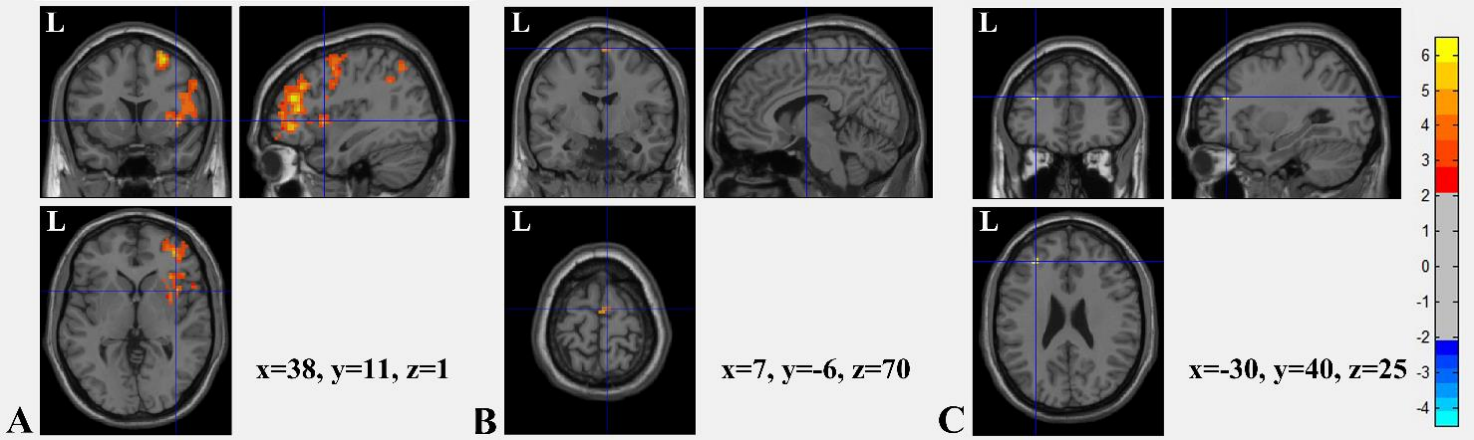
**T value graphs of two independent samples t-test for brain FC between two groups**. **(A)** With the right middle frontal gyrus as the ROI, the FC between the ROI and right insula (peak MNI coordinates: x=38, y=11, z=1) was found to be significantly decreased in the PD + CCPS group. **(B)** With the left insula as the ROI, the FC between the ROI and bilateral SMA (peak MNI coordinates: left: x=-1, y=-8, z=69; right: x=7, y=-6, z=70) decreased significantly in the PD + CCPS group. **(C)** With the right insula as the ROI, the FC between the ROI and left middle frontal gyrus (peak MNI coordinates: x=-30, y=40, z=25) decreased significantly in the PD + CCPS group. Color bar indicates the significance levels in the clusters in t values. (*p*<0.05, TFCE corrected) FC: functional connectivity; ROI: region of interest; SMA: supplementary motor area; MNI: Montreal Neurological Institute.

## RESULTS

### Demographic and clinical characteristics

Age, sex, BMI, years of education, disease duration, and MMSE and MoCA scores showed no significant differences between the two groups. The demographic features of subjects enrolled in gait analysis (Table 1) and those enrolled in fMRI analysis (Table 2) were also comparable.

The PD + CCPS group had lower BBS scores than the PD - CCPS group (*p*=0.002) and took longer to perform the TUG test (*p*=0.004), indicating that the PD + CCPS group had poorer balance control and overall walking ability (Table 3).

**Figure 2.**
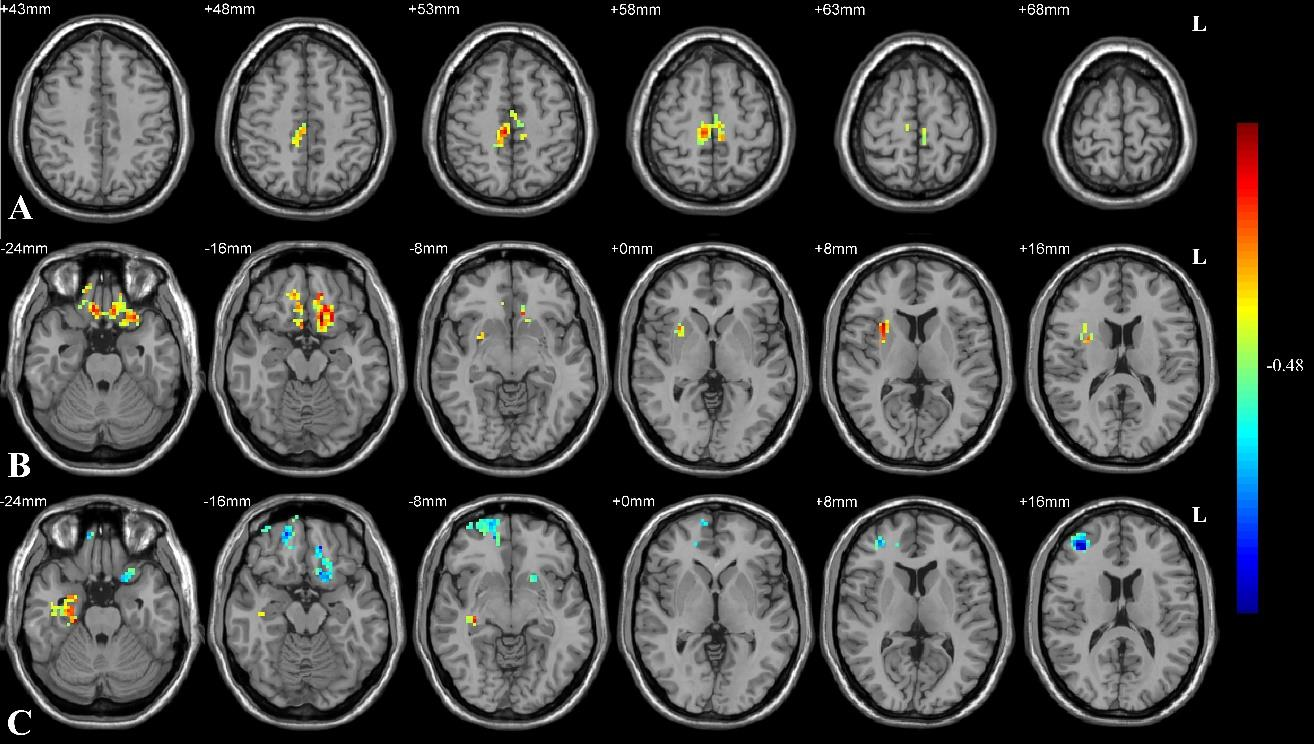
**Correlation between FC and gait parameters**. **(A)** Decreased FC between the left insula and bilateral SMA was significantly correlated with lower BBS score and (B) that between the right insula and bilateral middle frontal gyrus was correlated with decreased pelvic obliquity angle and (C) significantly longer time needed in the TUG test (*p*<0.05, AlphaSim corrected). The color bar indicates the correlation levels in the clusters in r values. FC: functional connectivity; SMA: supplementary motor area; BBS: Berg Balance Scale; TUG: Timed Up and Go Test.

**Table 3:** Gait parameters of PD patients.

	PD - CCPS (n=21)	PD + CCPS (n=16)	*p* value
**Gait-related scales**			
**BBS**	54.0(50.0, 55.0)^a^	43.5(40.3, 49.8)	0.002^b^
**TUG (s)**	10.3(8.5, 12.2)^a^	16.1(10.8, 18.5)	0.004^b^
**Kinematic parameters**			
**Cadence (steps/min)**	105.0±12.8	104.4±14.8	0.905^c^
**Double support time (s)**	0.24(0.21, 0.31)	0.25(0.22, 0.30)	0.660^b^
**Step time (s)**	0.6±0.1	0.6±0.1	0.937^c^
**Step length/height (m/m)**	0.32(0.30, 0.35)	0.30(0.26, 0.34)	0.059^b^
**Step width/height (m/m)**	0.09(0.07, 0.10)	0.09(0.06, 0.11)	0.774^b^
**Stride time (s)**	1.2±0.1	1.2±0.2	0.842^c^
**Stride length/height (m/m)**	0.64±0.07	0.58±0.13	0.068^c^
**Walking speed/height (/s)**	0.56±0.11	0.50±0.13	0.138^c^
**Dynamic parameters**			
**Pelvic tilt (°)**	19.0±7.1	22.2±9.5	0.256^c^
**Pelvic obliquity (°)**	5.3(4.4, 6.8)	3.7(3.0, 4.3)	0.004^b^
**Pelvic rotation (°)**	8.2(5.3, 9.5)	7.7(4.2, 13.0)	1.000^b^
**Knee flexion range (°)**	54.5±8.5	54.9±11.1	0.903^c^
**Ankle flexion range (°)**	29.7±5.3	29.7±5.7	0.989^c^
**CoM**			
**Lowering of CoM (yes/no, n)**	5/16	9/6^d^	0.028^e^

PD: Parkinson’s disease; CC: camptocormia; PS: Pisa syndrome; PD - CCPS: PD without CC or PS group; PD + CCPS: PD with CC or PS group; BBS: Berg Balance Scale; TUG: Timed Up and Go Test; CoM: center of mass. The values are expressed as the mean ± SD for normally distributed continuous variables, mean (interquartile range) for abnormally distributed continuous variables, and number for categorical variables.

a Data were unavailable for 8 patients.

b Mann-Whitney test.

c Independent sample t-test.

d Data were unavailable for 1 patient.

e Crosstabs. *p*<0.05 indicate statistical significance.

No significant differences in kinematic parameters were found between the two groups. In the dynamic parameters, the PD + CCPS group had a smaller angle of pelvic obliquity than the PD - CCPS group (3.7(3.0, 4.3)° vs. 5.3(4.4, 6.8)°, *p*=0.004), which showed that the former had a narrower range of motion for pelvic coronal tilt while walking. In addition, the proportion of patients with progressive downward CoM movement was significantly higher in the PD + CCPS group than in the PD - CCPS group (60.0% vs. 23.8%, *p*=0.028) (Table 3). Multivariate correlation analysis indicated that the pelvic obliquity angle (*p*=0.019, OR=0.458, 95%CI: 0.239-0.877) and the progressive downward movement of the CoM during walking (*p*=0.023, OR=8.897, 95%CI: 1.355-58.444) were independently associated with postural abnormalities in PD (Table 4).

### Discovering FC abnormalities in the PDPA group

After analyses of the insula and frontal lobes as ROIs, significant differences between the FC maps of the two groups were shown in the connections between the right middle frontal gyrus and right insula (peak MNI coordinates: x=38, y=11, z=1; right middle frontal gyrus as ROI, Fig. 1A), between the left insula and bilateral supplementary motor areas (SMA; peak MNI coordinates, left SMA: x=-1, y=-8, z=69; right SMA: x=7, y=-6, z=70; left insula as ROI, Fig. 1B), and between the right insula and left middle frontal gyrus (peak MNI coordinates: x=-30, y=40, z=25; right insula as ROI, Fig. 1C) (all *p* < 0.05, TFCE-corrected). These results indicated that the FC between the left insula and bilateral SMA and between the right insula and bilateral middle frontal gyrus were significantly weaker in the PD + CCPS group than in the PD - CCPS group.

### Confirming correlations between FC decreases and gait dysfunction

Gait parameters with significant differences, including BBS score, TUG test time, and pelvic obliquity angle, were selected for correlation analyses with FC in the areas mentioned above. The results showed that patients with low BBS scores had decreased FC between the left insula and bilateral SMA and that those with a small pelvic obliquity angle or poor TUG performance had decreased FC between the right insula and bilateral middle frontal gyrus (all *p* < 0.05, AlphaSim corrected) (Fig. 2).

**Table 4:** Independently associated factors within the gait parameters of PDPA.

	Univariate analysis			Multivariate analysis		
	*p* value	OR	95%CI	*p* value	OR	95%CI
**Pelvic obliquity (°)**	0.017	0.546	(0.332-0.899)	0.019	0.458	(0.239-0.877)
**Lowering of CoM (yes)**	0.033	4.800	(1.137-20.272)	0.023	8.897	(1.355-58.444)

CoM: center of mass; OR: odds ratio; CI: confidence interval. *p*<0.05 is considered statistically significant.

### Providing a structural basis for functional abnormalities via FA comparison

**Figure 3.**
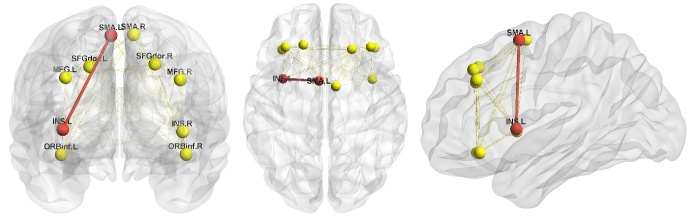
**Comparison of average FA between two groups**. The PD + CCPS group exhibited decreased structural connectivity compared with the PD - CCPS group in the fiber connecting the left insula and left SMA (red nodes and red thick lines; *p* = 0.019). No significant differences in the other fibers were found between the two groups (*p* > 0.05; yellow nodes and yellow thin lines). SMA.L: left supplementary motor area; SMA.R: right supplementary motor area; INS.L: left insula; INS.R: right insula; SFGdor.L: left dorsal superior frontal gyrus; SFGdor.R: right dorsal superior frontal gyrus; MFG.L: left middle frontal gyrus; MFG.R: right middle frontal gyrus; ORBinf.L: orbital part of left inferior frontal gyrus; ORBinf.R: orbital part of right inferior frontal gyrus.

As we found significant FC abnormalities with correlated gait dysfunctions between the left insula and bilateral SMA and between the right insula and bilateral middle frontal gyrus in the PDPA group, we further performed DTI structural connectivity analysis searching for a structural basis. The average FA of fibers between the left insula and bilateral SMA and of fibers between the right insula and bilateral middle frontal gyrus were analyzed. A significant average FA decrease in the fibers (*p* = 0.019) connecting the left insula and left SMA was found in the PD + CCPS group (Fig. 3). However, there were no significant differences in the other fibers between the two groups (*p* > 0.05).

## DISCUSSION

Pelvic movement is important for maintaining trunk posture and ambulatory limb movement. Testing of a gait assistance robot design based on a mouse model of severe spinal cord damage showed that the direction of the trunk is closely related to the movement and muscle activity of the lower limbs and receives balance correction from the proprioceptive sensory feedback of the flexor and extensor muscles, highlighting the importance of trunk adjustment in balance during walking [35]. A number of previously used nerve rehabilitation instruments fixed the pelvis during walking training, but some studies further found pelvic fixation to be unfavorable for gait dynamics, suggesting that pelvic fixation should be avoided in walking training [36]. In our study, PDPA was found to feature a significant decrease in pelvic obliquity angle, meaning a reduced coronal fluctuation range of the pelvis as well as its connections to the legs, which is similar to the pattern of pelvic fixation mentioned previously and thus inducing poorer gait dynamics.

In our 3D gait test, a small number of subjects with normal posture were observed to have a slight but significant progressive downward movement of the CoM, which neither the patients nor the neurologists detected visually. Moreover, our study indicated that the proportion of this phenomenon was significantly higher in PDPA than in PD with normal posture. No other studies use this indicator for reference. The pathogenesis of PDPA is likely a process from quantitative change to qualitative change; therefore, the progressive downward movement of the CoM during walking, as detected by the 3D gait test, could potentially be an early predictor of CC or PS in PD. This parameter is worth exploring in other diseases, such as multiple system atrophy, which also commonly co-occurs with postural abnormalities. However, prospective studies are needed to provide higher levels of clinical evidence of its predictive efficacy.

The insula is generally believed to be closely related to motor control and cognitive function. A study on chronic subjective dizziness (CSD) using fMRI demonstrated that the insula, as part of the parieto-insular vestibular cortex (PIVC), has significantly weaker FC with the rest of the PIVC in CSD patients than in healthy individuals, which may be related to the long-term vestibular symptoms of CSD[11]. Thus, the insula is important for the vestibular function of the human body; on the other hand, vestibular dysfunction is part of the pathogenic mechanism of postural abnormalities in PD. Furthermore, our study shows that the FC of the insula with several other areas in the brain decreases in PDPA. All these results point to the noteworthy relationship between the insula and pathogenic mechanism of PDPA.

The frontal lobe has been shown to be important in postural adjustment and CoM stability in a study using electroencephalography [37]. Another study found that the ability to imitate finger gestures is related to the frontal lobe, insula and basal ganglia in the brain functional network of apraxia [13]. On the one hand, apraxia may share some mechanisms with PDPA, considering their similar clinical characteristics. On the other hand, the postures studied in apraxia fall within the category of limbs rather than the trunk, but common mechanisms likely exist for the postural abnormality of different parts in the human body. In our study, the FC connecting the insula with both the middle frontal gyrus and SMA decreased significantly in PDPA, as did the FA of the fiber connecting the left insula and left SMA, further indicating that the frontal lobe, SMA and insula are related to postural abnormalities in PD. In addition, other clinical studies have shown that PDPA patients also have other notable clinical symptoms of frontal lobe dysfunctions, such as fatigue, attentional decline, and memory deficit [38]. These results provide evidence for the relationship between the frontal lobe (especially the middle frontal gyrus), SMA, insula and PDPA and provide evidence for further basic research and potential intervention.

Previous studies have shown that the frontal lobe, the insula combined with the parietal lobe, and the anterior cingulate gyrus play an important role in goal-directed movements. Additionally, the frontal lobe, SMA, and cerebellar vermis work together to control postural and motor stability when movement involves acceleration and balance [39]. A study that used event-related functional near-infrared spectroscopy to explore the mechanism of balance disabilities in hemiplegic stroke patients revealed that oxygenated hemoglobin levels of bilateral prefrontal lobes and bilateral SMA regions are positively correlated with BBS balance ability [40]. In our study, decreased FC between the left insula and bilateral SMA, correlated with decreased BBS balance ability and decreased FA between the left insula and left SMA, was shown to exist in PDPA, which is consistent with previous studies in which SMA was reported to regulate balance ability. Moreover, our study finds that weakness of FC between the right insula and bilateral middle frontal gyrus is related to decreases in the coronal motor range of the pelvis and comprehensive walking ability in the TUG test, which is also consistent with previous studies confirming the effect of the frontal lobe and insula on goal-targeted movement. Thus, weakness of FC between the insula, SMA and frontal lobes with a decrease in structural connectivity between the left insula and left SMA in PDPA is probably the internal mechanism of its coexisting gait dysfunction.

Our study has some limitations. The observed gait parameters in this study include only those closely related to clinical practice; other potentially valuable indicators may have been omitted. Additionally, as a retrospective case-control study, our study cannot address the sequential or causal relationships of the correlated results. Moreover, a larger sample size would increase the accuracy and credibility of the results. In our study, most PDPA patients suffered advanced stages of PD, and motor ability was severely limited in the off-state; therefore, we performed all assessments 1-2 h after the subjects took their routine medication. We admit that if feasible and safe, off-state study would further avoid potential interference of medication effects and may provide more accurate evidence of pathogenic exploration. Additionally, the MRI analysis may have produced some false positive or false negative results, although such errors have been minimized by cross-referencing the statistical findings for MRI data and clinical manifestations in our study.

In conclusion, this study is the first to show that a decreased pelvic obliquity angle and progressive downward movement of the CoM during walking are independently associated with postural abnormalities in PD. Additionally, we have demonstrated that gait dysfunction is correlated with decreased FC between the left insula and bilateral SMA and between the right insula and bilateral middle frontal gyrus; additionally, decreased FA of the fibers connecting the left insula with the left SMA provides structural evidence for the underlying pathogenic mechanism of PDPA. These findings provide a new reference for the underlying pathogenesis and early detection of postural abnormalities in PD and suggest new targets for potential intervention.

## References

[1] Doherty KM, van de Warrenburg BP, Peralta MC, Silveira-Moriyama L, Azulay JP, Gershanik OS, et al (2011). Postural deformities in Parkinson's disease. Lancet Neurol, 10:538-549. 21514890 10.1016/S1474-4422(11)70067-9

[2] Ponfick M, Gdynia HJ, Ludolph AC, Kassubek J (2011). Camptocormia in Parkinson's disease: a review of the literature. Neurodegener Dis, 8:283-288. 21389682 10.1159/000324372

[3] Barone P, Santangelo G, Amboni M, Pellecchia MT, Vitale C (2016). Pisa syndrome in Parkinson's disease and parkinsonism: clinical features, pathophysiology, and treatment. Lancet Neurology, 15:1063-1074. 27571158 10.1016/S1474-4422(16)30173-9

[4] Nakane S, Yoshioka M, Oda N, Tani T, Chida K, Suzuki M, et al (2015). The characteristics of camptocormia in patients with Parkinson's disease: A large cross-sectional multicenter study in Japan. J Neurol Sci, 358:299-303. 26428310 10.1016/j.jns.2015.09.015

[5] Srivanitchapoom P, Hallett M (2016). Camptocormia in Parkinson's disease: definition, epidemiology, pathogenesis and treatment modalities. J Neurol Neurosurg Psychiatry, 87:75-85. 25896683 10.1136/jnnp-2014-310049 PMC5582594

[6] Vorovenci RJ, Biundo R, Antonini A (2016). Therapy-resistant symptoms in Parkinson's disease. J Neural Transm (Vienna), 123:19-30. 26410626 10.1007/s00702-015-1463-8

[7] Bonneville F, Bloch F, Kurys E, du Montcel ST, Welter ML, Bonnet AM, et al (2008). Camptocormia and Parkinson's disease: MR imaging. Eur Radiol, 18:1710-1719. 18351343 10.1007/s00330-008-0927-8

[8] Frazzitta G, Balbi P, Gotti F, Maestri R, Sabetta A, Caremani L, et al (2015). Pisa Syndrome in Parkinson's Disease: Electromyographic Aspects and Implications for Rehabilitation. Parkinsons Disease. 10.1155/2015/437190 PMC4670865 26682083

[9] Tinazzi M, Juergenson I, Squintani G, Vattemi G, Montemezzi S, Censi D, et al (2013). Pisa syndrome in Parkinson's disease: an electrophysiological and imaging study. Journal of Neurology, 260:2138-2148. 23695587 10.1007/s00415-013-6945-8

[10] Margraf NG, Rohr A, Granert O, Hampel J, Drews A, Deuschl G (2015). MRI of lumbar trunk muscles in patients with Parkinson's disease and camptocormia. J Neurol, 262:1655-1664. 25929656 10.1007/s00415-015-7726-3

[11] Indovina I, Riccelli R, Chiarella G, Petrolo C, Augimeri A, Giofre L, et al (2015). Role of the Insula and Vestibular System in Patients with Chronic Subjective Dizziness: An fMRI Study Using Sound-Evoked Vestibular Stimulation. Front Behav Neurosci, 9:334. 26696853 10.3389/fnbeh.2015.00334 PMC4673311

[12] Gallea C, Ewenczyk C, Degos B, Welter ML, Grabli D, Leu-Semenescu S, et al (2017). Pedunculopontine network dysfunction in Parkinson's disease with postural control and sleep disorders. Mov Disord, 32:693-704. 28164375 10.1002/mds.26923

[13] Lesourd M, Osiurak F, Baumard J, Bartolo A, Vanbellingen T, Reynaud E (2018). Cerebral correlates of imitation of intransitive gestures: An integrative review of neuroimaging data and brain lesion studies. Neurosci Biobehav Rev, 95:44-60. 30086324 10.1016/j.neubiorev.2018.07.019

[14] Tramonti C, Di Martino S, Unti E, Frosini D, Bonuccelli U, Rossi B, et al (2017). Gait dynamics in Pisa syndrome and Camptocormia: The role of stride length and hip kinematics. Gait Posture, 57:130-135. 28623760 10.1016/j.gaitpost.2017.05.029

[15] Barkhof F, Haller S, Rombouts SA (2014). Resting-state functional MR imaging: a new window to the brain. Radiology, 272:29-49. 24956047 10.1148/radiol.14132388

[16] Fjell AM, Westlye LT, Greve DN, Fischl B, Benner T, van der Kouwe AJ, et al (2008). The relationship between diffusion tensor imaging and volumetry as measures of white matter properties. Neuroimage, 42:1654-1668. 18620064 10.1016/j.neuroimage.2008.06.005 PMC2808804

[17] Postuma RB, Berg D, Stern M, Poewe W, Olanow CW, Oertel W, et al (2015). MDS clinical diagnostic criteria for Parkinson's disease. Mov Disord, 30:1591-1601. 26474316 10.1002/mds.26424

[18] Ou R, Liu H, Hou Y, Song W, Cao B, Wei Q, et al (2018). Predictors of camptocormia in patients with Parkinson's disease: A prospective study from southwest China. Parkinsonism Relat Disord, 52:69-75. 29606604 10.1016/j.parkreldis.2018.03.020

[19] Huh YE, Kim K, Chung WH, Youn J, Kim S, Cho JW (2018). Pisa Syndrome in Parkinson's Disease: Pathogenic Roles of Verticality Perception Deficits. Scientific Reports, 8. 29379091 10.1038/s41598-018-20129-2 PMC5788854

[20] Goetz CG, Tilley BC, Shaftman SR, Stebbins GT, Fahn S, Martinez-Martin P, et al (2008). Movement Disorder Society-sponsored revision of the Unified Parkinson's Disease Rating Scale (MDS-UPDRS): scale presentation and clinimetric testing results. Mov Disord, 23:2129-2170. 19025984 10.1002/mds.22340

[21] Hoehn MM, Yahr MD (1967). Parkinsonism: onset, progression and mortality. Neurology, 17:427-442. 6067254 10.1212/wnl.17.5.427

[22] Folstein MF, Folstein SE, McHugh PR (1975). "Mini-mental state". A practical method for grading the cognitive state of patients for the clinician. J Psychiatr Res, 12:189-198. 1202204 10.1016/0022-3956(75)90026-6

[23] Nasreddine ZS, Phillips NA, Bedirian V, Charbonneau S, Whitehead V, Collin I, et al (2005). The Montreal Cognitive Assessment, MoCA: a brief screening tool for mild cognitive impairment. J Am Geriatr Soc, 53:695-699. 15817019 10.1111/j.1532-5415.2005.53221.x

[24] Berg KO, Wood-Dauphinee SL, Williams JI, Maki B (1992). Measuring balance in the elderly: validation of an instrument. Can J Public Health, 83 Suppl 2:S7-11. 1468055

[25] Podsiadlo D, Richardson S (1991). The timed "Up & Go": a test of basic functional mobility for frail elderly persons. J Am Geriatr Soc, 39:142-148. 1991946 10.1111/j.1532-5415.1991.tb01616.x

[26] Wang J, Wang X, Xia M, Liao X, Evans A, He Y (2015). GRETNA: a graph theoretical network analysis toolbox for imaging connectomics. Front Hum Neurosci, 9:386. 26175682 10.3389/fnhum.2015.00386 PMC4485071

[27] Lo OY, Halko MA, Zhou J, Harrison R, Lipsitz LA, Manor B (2017). Gait Speed and Gait Variability Are Associated with Different Functional Brain Networks. Front Aging Neurosci, 9:390. 29249961 10.3389/fnagi.2017.00390 PMC5715372

[28] Tzourio-Mazoyer N, Landeau B, Papathanassiou D, Crivello F, Etard O, Delcroix N, et al (2002). Automated anatomical labeling of activations in SPM using a macroscopic anatomical parcellation of the MNI MRI single-subject brain. Neuroimage, 15:273-289. 11771995 10.1006/nimg.2001.0978

[29] Yan CG, Wang XD, Zuo XN, Zang YF (2016). DPABI: Data Processing & Analysis for (Resting-State) Brain Imaging. Neuroinformatics, 14:339-351. 27075850 10.1007/s12021-016-9299-4

[30] Song XW, Dong ZY, Long XY, Li SF, Zuo XN, Zhu CZ, et al (2011). REST: a toolkit for resting-state functional magnetic resonance imaging data processing. PLoS One, 6:e25031. 21949842 10.1371/journal.pone.0025031 PMC3176805

[31] Smith SM (2002). Fast robust automated brain extraction. Hum Brain Mapp, 17:143-155. 12391568 10.1002/hbm.10062 PMC6871816

[32] Andersson JLR, Sotiropoulos SN (2016). An integrated approach to correction for off-resonance effects and subject movement in diffusion MR imaging. Neuroimage, 125:1063-1078. 26481672 10.1016/j.neuroimage.2015.10.019 PMC4692656

[33] Cui Z, Zhong S, Xu P, He Y, Gong G (2013). PANDA: a pipeline toolbox for analyzing brain diffusion images. Front Hum Neurosci, 7:42. 23439846 10.3389/fnhum.2013.00042 PMC3578208

[34] Eloy M-H, Federico V, Vesna P, Carlos L, Magí A, Elena HM-L, et al 2015 Deterministic DTI tractography based on fiber assignement by continuous tracking (FACT) in ten healthy subjects in two HARDI datasets.

[35] Moraud EM, von Zitzewitz J, Miehlbradt J, Wurth S, Formento E, DiGiovanna J, et al (2018). Closed-loop control of trunk posture improves locomotion through the regulation of leg proprioceptive feedback after spinal cord injury. Sci Rep, 8:76. 29311614 10.1038/s41598-017-18293-y PMC5758718

[36] Veneman JF, Menger J, van Asseldonk EH, van der Helm FC, van der Kooij H (2008). Fixating the pelvis in the horizontal plane affects gait characteristics. Gait Posture, 28:157-163. 18207406 10.1016/j.gaitpost.2007.11.008

[37] Varghese JP, Merino DM, Beyer KB, McIlroy WE (2016). Cortical control of anticipatory postural adjustments prior to stepping. Neuroscience, 313:99-109. 26608123 10.1016/j.neuroscience.2015.11.032

[38] Abe K, Uchida Y, Notani M (2010). Camptocormia in Parkinson's disease. Parkinsons Dis, 2010. 10.4061/2010/267640 PMC2951140 20948888

[39] Hamacher D, Herold F, Wiegel P, Hamacher D, Schega L (2015). Brain activity during walking: A systematic review. Neurosci Biobehav Rev, 57:310-327. 26306029 10.1016/j.neubiorev.2015.08.002

[40] Mihara M, Miyai I, Hattori N, Hatakenaka M, Yagura H, Kawano T, et al (2012). Cortical control of postural balance in patients with hemiplegic stroke. Neuroreport, 23:314-319. 22357394 10.1097/WNR.0b013e328351757b

